# Pituitary-Adrenal Axis in Prader Willi Syndrome

**DOI:** 10.3390/diseases4010005

**Published:** 2016-01-19

**Authors:** Olivia S. Edgar, Angela K. Lucas-Herald, Mohamad Guftar Shaikh

**Affiliations:** Developmental Endocrinology Research Group, Royal Hospital for Children, University of Glasgow, 1345 Govan Road, Glasgow G41 4TF, UK; 1107167e@student.gla.ac.uk (O.S.E.); angelalh@doctors.org.uk (A.K.L.-H.)

**Keywords:** adrenal, steroid, hydrocortisone, PWS

## Abstract

Purpose: Prader Willi syndrome (PWS) is a rare genetic condition that has concurrent endocrinological insufficiencies. The presence of growth hormone deficiency has been well documented, but adrenal insufficiency (AI) is not widely reported. A review was conducted to investigate its prevalence and relevance in PWS in both adults and children. Methodology: A literature review was performed with the search terms “Prader-Willi syndrome” and “adrenal insufficiency”. Results: The review found studies disagree on the prevalence and method of investigation of AI in PWS. Case studies demonstrate that patients with PWS are at risk of premature death, often secondary to respiratory infections. The possibility that this may be the result of the inability to mount an effective cortisol response has been studied, with some evidence confirming AI in PWS patients. Most reports agreed AI is present in PWS, however, Farholt *et al.* showed no HPA axis dysfunction in adults, suggesting that perhaps it is rare in adults, and children should be the focus of further studies. Conclusion: AI is present in some patients with PWS. Further research is required to ensure optimal treatment can be implemented and to prevent premature deaths related to adrenal insufficiency. Clinicians should have a low threshold for testing the adrenal axis and considering treatment for adrenal insufficiency in PWS patients.

## 1. Introduction

Prader Willi syndrome (PWS) was first described in 1956 [1]. It is a genetic condition, usually secondary to a deletion of a segment of the paternally-inherited chromosome 15q11-q13, although other genetic abnormalities have been described, including maternal uniparental disomy of the same chromosome [2]. PWS is rare with a UK prevalence estimated to be around 1:52,000 and a birth incidence of approximately 1:29,000 [3]. The prevalence appears to be rising but this could be due to increasing awareness of the syndrome and increased diagnosis in paediatric care [4].

The different manifestations of PWS vary with age, but not gender [5]. Affected infants initially present with feeding problems due to hypotonia and a poor suck, and the condition, in fact, represents 11% of children under the age of two years presenting with hypotonia [6]. From the age of two years the clinical features are dominated by increasing weight gain, hyperphagia, and an insatiable appetite. Other features include short stature, learning difficulties, dysmorphic facial features, low lean body mass, hypogonadism, and sleep and behavioural disturbance [2,7]. The endocrine features of PWS are well recognised and can include Growth Hormone Deficiency (GHD) or insufficiency, cryptorchidism and hypogonadism, hypothyroidism, impaired glucose tolerance and diabetes mellitus [2]. Short term treatment with recombinant growth hormone (GH) for children with PWS was first reported in 1987 and PWS has since become a licensed indication for its use [8].

A less well studied aspect of PWS is the occurrence of adrenal insufficiency (AI). AI is the impaired production of cortisol or action of ACTH, including unresponsiveness of the adrenals to ACTH [9]. Although AI was not included in the original descriptions of PWS, more recent studies have proposed it as a possible additional feature.

To determine the importance of AI in patients with PWS, we performed a literature search with Pubmed and Google Scholar using the terms “Prader Willi syndrome” and “adrenal insufficiency” (AI), with the aim to consider current literature regarding the frequency and significance of adrenal insufficiency in patients with PWS.

## 2. Why Could AI Be an Issue in PWS?

Studies have demonstrated that PWS is associated with a high rate of sudden unexpected death at 3% [3,10], although these have been limited by their small sample size. As GH therapy has been introduced in recent years, there have been some concerns that there could be an association between GH therapy and sudden unexplained death in PWS. Nagai *et al.* [11], therefore, performed a *post mortem* study on 13 patients who had not received GH therapy and seven who had. They found that cause of death tended to correlate with age and stage of development rather than with GH treatment. Young infants (0–1 year) typically died of milk aspiration secondary to hypothalamic dysregulation of respiratory function. Young children and adolescents (1–19 years) died from complications of respiratory infections, while young adults (≥20 years) died from the complications of obesity and diabetes mellitus [11]. Tauber *et al.* [12] undertook a similar study, but had a larger cohort of 64 patients. Again, respiratory disorders were the most common cause of death (COD) (61%) and GH therapy had no correlation with increased death rate [12]. Table 1 summarises papers which report the cause of death in patients with PWS.

**Table 1:** Cause of death in patients with PWS-summary of reports.

Author(s)	Patients	Method	Results: Cause of Death
Schrander-Stumpel *et al.*, 2004 [13]	27 PWS patients who died (age range birth–8 years)	Case reports reviewed after death	Four cases of early morning sudden unexplained death in context of infectious symptoms
Stevenson *et al.*, 2004 [14]	Eight patients with PWS who died, ages 5 months–42 years	Case reports reviewed after death	Three patients had hypoplastic adrenal glands at time of postmortem
Van Vliet *et al.*, 2004 [15]	1 PWS patient who died aged four years	Case report reviewed after death	Likely sleep apnoea and tonsillar hypertrophy resulting in sudden death
Vogels *et al.*, 2004 [4]	78 patients with PWS (mean age 26 (range 0–56 years))—seven died	Data collected from genetic centers in Flanders	Sudden respiratory infections with a high temperature most common. 1/7 (14%) patient died of sudden collapse—cause unknown
Nagai *et al.*, 2005 [11]	13 PWS patients who had received GH therapy and seven who had not	*Post mortem* study	Dependent on age group; young infants (milk aspiration due dysregulation of respiratory function). Young children (complications of respiratory infections). Teenagers and young adults (complications of obesity and diabetes)
Tauber *et al.*, 2008 [12]	64 patients (age range 1 day–19 years), 28 on GH therapy	Study of *post mortem* reports	Respiratory causes in 24 (63%) including infection and obstruction secondary to tonsillar hypertrophy. Sudden death of unknown cause in 11 (29%)

While the above studies establish a common primary cause of death, they do not fully explore any underlying abnormality which could cause PWS children to be more susceptible to these life-threatening respiratory infections. One possible explanation is an inability to mount an effective cortisol response [10,14]. This was suggested after a *post mortem* study on eight individuals with PWS (four infants aged 5–19 months, two toddlers aged 3.5 years, and two adults aged 29 and 43 years) [14]. Four of these individuals had an autopsy that recorded adrenal weight and of these three had well below average adrenal weight for body length. This theorises that there could be an unrecognised peripheral adrenal insufficiency in PWS children perhaps contributing to their deaths [14]. A larger cohort of patients for further studies would be needed, however, to confirm this association.

To further investigate this hypothesis, one study used 3D MRI to review the pituitary gland while simultaneously measuring basal pituitary hormonal levels in patients with PWS [16]. This found a high prevalence of morphological pituitary abnormalities, such as hypoplasia and universal anterior hormone deficiencies in PWS subjects. Although they did not measure ACTH specifically, if other anterior hormones were deficient it is possible that ACTH would be too, although the authors recognise that further studies are needed to explore this [16]. Interestingly this study had both a control group and a group with early onset morbid obesity (EMO). The patients with EMO showed very similar results to those with PWS, which could indicate that the aetiology of this pituitary deficiency may be, in part, secondary to the obesity phenotype of PWS rather than congenital in nature, or may be just normal variation.

In an attempt to explain the high sudden unexpected death rate in children with PWS, studies [3,10] have postulated that PWS patients may be unable to mount an appropriate cortisol response during stressful conditions, such as infection. Physiological stress, often induced by critical illness, results in an acute and prolonged response of the HPA axis, causing an increase in plasma cortisol and ACTH levels [17].

In 2008, de Lind van Wjingarden *et al.* [10] studied 25 randomly-selected PWS patients in a paediatric intensive care unit (PICU), and used overnight single-dose metyrapone testing to measure ACTH response alongside 11-deoxycortisol, cortisol and glucose levels. The median age was 9.7 (range 6.8–13.6) years. Diurnal salivary cortisol profiles were also assessed on an alternative day to when the patients were receiving metyrapone. Central AI was confirmed if the ACTH response was 33 pmol/L at 0730 h. They found that 15 patients (60%) fulfilled the criteria of AI, however salivary cortisol levels were normal in all patients, suggesting that AI only became apparent in induced stressful conditions [10] and might offer an explanation for the high death rate from stress-related illnesses.

Following this study, two further studies were carried out to see if this level of AI was replicated [18,19]. Corrias reviewed the adrenal responses of 84 PWS patients in a tertiary referral centre. A total of 35 females and 49 males (age range 0.8–17.9 years) were enrolled in the study. The adrenal response of these patients was assessed by measuring morning cortisol and ACTH dosage, using a 1 µg synacthen test or low dose short synacthen test (LDSST), and an appropriate response was considered to be more than 500 nmol/L. An LDSST supplies a lower dose of synthetic ACTH than a standard short synacthen test (SST) where 250 micrograms of ACTH is administered. The LDSST, therefore, represents the adrenal cortical response to ACTH administration, with studies suggesting it may be the most reliable test for ACTH deficiency [20,21] as the LDSST may detect adrenal insufficiency in those who had a normal SST [22], although this is a controversial area as there is no clear evidence that one test is superior to the other. On the low dose synacthen stimulation test 12 patients had unsatisfactory cortisol responses (14.3%) with reduced basal (169.4 ± 83.3 nmol/L) and stimulated (428.1 ± 69.6 nmol/L) cortisol levels [18]. These patients were retested using a standard dose 250 µg synacthen test which confirmed AI in four patients (4.8%). This result agreed with the study by de Lind van Wjingarden *et al.* that AI is present in some PWS children. However, the statistics showed it was less prevalent than previously reported [10].

A similar study was conducted as a follow up to the de Lind van Wjingarden paper by Nyunt *et al.* [19]. This group used a low dose synacthen test (LDSST) (1 µg) to screen for AI in 41 PWS children (mean age 7.68 (±5.23)) years, and measured ACTH and cortisol levels. This study, however, provided contradictory evidence, as all children demonstrated a normal cortisol response of ≥500 nmol/L [19].

Further contradictory evidence was provided in a study by Farholt *et al.* Their cohort consisted of 57 participants, 18 under the age of 17 and 39 adults (total median age 22; age range 0.6–48) and, of this cohort, none showed any HPA dysfunction in response to a standard high dose synacthen test or insulin tolerance test. From this we could conclude that not only is this condition rare but perhaps a paediatric issue [23].

Another study that used LDSST on 53 PWS adults to determine the presence of AI [23] showed that eight participants (15.1%) had an insufficient cortisol response, with a peak cortisol of <500 nmol/L. These subjects were retested with a high dose short synacthen test (HDSST) and, of the eight, four (7.5%) had suboptimal responses [24].

The above studies are summarised in Table 2. There is no obvious explanation for the differing results, however the authors have suggested that perhaps the differing test methods or cut off levels could be responsible. Measurement of cortisol using different assays itself has also changed over time with lowering of acceptable cortisol thresholds. There is no current gold standard in terms of the type of test used for AI in PWS or the frequency of monitoring required. To truly understand the prevalence of AI in PWS children, further studies must be done.

**Table 2:** Summary of studies of adrenal insufficiency in PWS.

Author(s)	Patient Group	Method	Level Considered Sufficient	Results
Grugni *et al.*, 2013 [24]	53 PWS adults	Low dose short synacthen test. Deficient patients retested with high dose short synacthen test	Peak cortisol response >500 nmol/L	After LDSST eight (15.1%) had a PCR of <500 nmol/L. On retesting 4 showed persistent suboptimal response (7.5%)
Corrias *et al.*, 2012 [18]	84 PWS children (35 females and 49 males aged 0.8–17.9 years).	Low-dose tetracosactrin test 1 µg and standard dose tetracosactrin test (250 µg) in second round of testing	Peak cortisol response >500 nmol/L	12 patients (14.3%) had AI according to LDSST but when retested with SDSST only 4 (4.8%) were confirmed AI
Nyunt *et al.*, 2010 [19]	Randomly selected 41 PWS children (mean age 7.68 (±5.23) years)	Low dose synacthen test (1 µg)	Peak cortisol response >500 nmol/L	All children had a 30 min cortisol >500 nmol/L
De Lind van Wijngaarden *et al.*, 2008 [10]	25 randomly selected PICU patients, median age 9.7 (6.8–13.6 years)	Overnight single dose (30 mg/kg) metyrapone test	ACTH >33 pmol/L at 0730 h	Fifteen patients (60%) had an insufficient ACTH response
Farholt *et al.*, 2011 [23]	57 patients with PWS (mean age 22 years, range 0.5–48 years)	Standard high dose synacthen test or insulin tolerance test	Peak cortisol response>500 nmol/L. Rise in cortisol by 250nmol/L	All patients had a normal response to synacthen testing

## 3. The Effects of GH on AI in PWS

Adrenal insufficiency could also be iatrogenic in origin. Disruption of the HPA axis and growth hormone deficiency in PWS have been well documented with growth hormone therapy now in widespread use [25,26]. The AACE (American Association of Clinical Endocrinologists) guidelines for the use of GH therapy remind clinicians that GH can inhibit the enzyme 11 β-hydroxysteroid dehydrogenase type 1 causing a shift in cortisol metabolism to favour cortisone production and, therefore, reduce global cortisol levels [27,28]. This may not have any clinical effect on most patients but it may unmask central hypothyroidism and hypoadrenalism. [29]. They recommend, therefore, that patients who become hypoadrenal on initiation of therapy should receive glucocorticoid replacement.

Finally, it is known that GHD is associated with the development of other pituitary hormone deficiencies, including ACTH deficiency [30]. Surveillance for this is therefore recommended in cases of isolated GHD or cases with multiple pituitary hormone defects [30], suggesting that this should also be considered in patients with PWS.

## 4. Summary and Conclusions

Prader Willi syndrome is a congenital condition with a complex phenotype. It is clear that some association between PWS and AI appears to exist, although this may be a predominantly paediatric issue, as Farholt *et al*. demonstrated in 39 adult PWS subjects with a normal HPA axis [23]. Which PWS patients are most at risk of this remains unclear, as does how best to test for this worrying phenomenon. Having reviewed the current literature, we would advise that there is enough of a reported association for clinicians to have a low threshold for testing PWS for adrenal insufficiency and to treat according to the results and clinical picture. If there is clinical suspicion, it would be safer to treat with hydrocortisone, even if only during intercurrent illnesses or if severely unwell. Further research is required, however, to confirm what the mechanism behind AI is and which patients are most at risk and how best to monitor for this.
